# Polymyxin B-induced acute kidney injury in a burn patient: a case report and pharmaceutical care

**DOI:** 10.3389/fmed.2026.1817208

**Published:** 2026-05-07

**Authors:** Shanshan Ding

**Affiliations:** Department of Clinical Pharmacy, Jinan Central Hospital, Jinan, China

**Keywords:** burns, case report, kidney injury, pharmaceutical care, polymyxin B

## Abstract

**Background:**

Polymyxin B is increasingly used against multidrug-resistant Gram-negative bacteria but carries a risk of acute kidney injury (AKI). Burn patients are particularly vulnerable.

**Case summary:**

A 44-year-old male with 66% TBSA burns and sepsis received polymyxin B (50 mg q12h, then escalated to 100 mg q12h). Serum creatinine rose from 59 to 163 μmol/L within 3 days, meeting KDIGO AKI criteria. No other nephrotoxic agents were used. The Naranjo score was 5, indicating probable polymyxin B-induced AKI. After dose reduction to 50 mg q12h, renal function improved without discontinuing therapy.

**Conclusion:**

In burn patients, early renal function monitoring (within 3 days) and dose adjustment based on infection response and renal biomarkers are critical. Therapeutic drug monitoring is recommended when available.

## 1 Introduction

Polymyxin B was first introduced into clinical practice in 1959 for the treatment of Gram-negative bacterial infections. However, due to its pronounced nephrotoxicity, it was later supplanted by other safer antimicrobial agents (1, 2). In recent years, the escalating challenge of antimicrobial resistance, marked by the increasing prevalence of multidrug-resistant (MDR) and extensively drug-resistant (XDR) Gram-negative bacteria, has posed a formidable threat to human health and presented immense clinical challenges. This has led to a resurgence of polymyxins in clinical practice (2–6). Polymyxin B is a cyclic peptide antibiotic that acts by increasing the permeability of the bacterial cell membrane, neutralizing endotoxin, and promoting bacterial lysis (7). Compared with other polymyxins, polymyxin B is preferred for the treatment of invasive infections caused by carbapenem-resistant Gram-negative bacteria, due to its more favorable pharmacokinetic profile and lower risk of nephrotoxicity (8). Nevertheless, careful monitoring for renal toxicity is still required in the clinical setting (6). Burn patients are at high risk for AKI. Therefore, when polymyxin B is used in these patients, who are at increased risk for AKI, vigilant monitoring, early identification of toxicity, and prompt intervention should be implemented to ensure clinical medication safety.

## 2 Case presentation

A 44-year-old male patient, weighing 79 kg, previously healthy, was transferred to our hospital on June 20, 13 days after sustaining a high-voltage electrical injury. He was febrile (40.2 °C) with heart rate 116 beats/min, respiratory rate 26 breaths/min, a blood pressure of 125/55 mmHg. He was mechanically ventilated, with a nasogastric tube, a central venous line and an arterial line in place. He exhibited diminished responses to pain, temperature, and touch along with reduced limb muscle strength. Burn wounds involving approximately 66% of his total body surface area (TBSA) were present on his limbs and trunk, covered with necrotic tissue and purulent exudate. The patient was diagnosed with a major burn involving 66% TBSA (mixed II°–III°in depth), complicated by sepsis and pulmonary infection, along with an inhalation injury.

Relevant tests were completed. Complete Blood Count (CBC) showed a white blood cell (WBC) count of 11.81 × 10^9/L with a neutrophil percentage (NEUT%) of 92.3%, and a platelet count (PLT) of 209 × 10^9/L. The procalcitonin (PCT) level was elevated at 5.557 ng/mL. Liver and renal function tests revealed as the following: alanine aminotransferase (ALT) at 27 U/L, aspartate aminotransferase (AST) at 49 U/L, albumin at 27.7 g/L, blood urea nitrogen (BUN) at 15.4 mmol/L, and creatinine (Cr) at 86 μmol/L. Treatments included fluid resuscitation, maintenance of electrolyte and acid–base balance, wound debridement and dressing changes, tracheostomy with mechanical ventilation, replacement of venous catheter, and administration of ulinastatin, human albumin, roxatidine, magnesium isoglycyrrhizinate, ambroxol hydrochloride, etc. Budesonide suspension was given via nebulization to improve airway inflammation, meropenem for anti-infective therapy, diclofenac sodium suppository for antipyresis, and enteral nutrition suspension was initiated. On June 21, polymyxin B (50 mg IV q12h, equivalent to 500,000 IU) was added.

**Figure 1:**
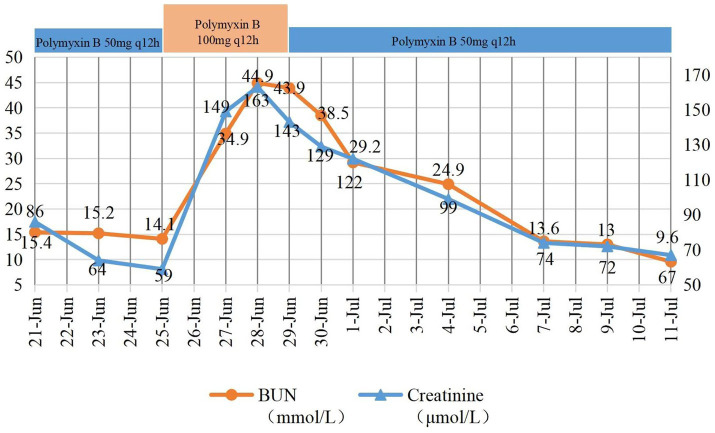
Patient’s renal function changes and polymyxin B medication regimen. BUN: blood urea nitrogen.

On June 25, the patient remained febrile (max 40.3 °C), with WBC 15.85 × 10^9/L, Albumin 33.8 g/L, BUN 14.1 mmol/L, creatinine 59 μmol/L. Wound secretion and sputum cultures grew multidrug-resistant *Acinetobacter baumannii*, which was susceptible to tigecycline and colistin. Based on the patient’s overall clinical status and in accordance with pertinent guideline recommendations, the antimicrobial regimen was adjusted to polymyxin B (100 mg IV q12h) in combination with tigecycline (50 mg IV q12h). On June 28, the patient’s body temperature decreased to 37.2 °C, with a PCT of 1.911 ng/mL. The patient’s creatinine and BUN levels had increased, with a BUN of 44.9 mmol/L and creatinine of 163 μmol/L. Following discussion between the attending physician and the clinical pharmacist, the decision was made to continue polymyxin B therapy based on improving infection markers and microbiological findings, with a reduction of the polymyxin B dose to 50 mg per dose beginning June 29. Tigecycline was continued (50 mg q12h) to exert a synergistic effect with polymyxin B against CRAB to improve treatment efficacy. Other nephrotoxic agents were avoided during this period. A monitoring plan was established, including renal function (Cr, BUN) monitored daily; urine output monitored every 6 h, and AKI defined per KDIGO criteria. The patient’s daily urine output was around 4,000 mL. On July 1, the patient’s renal function improved, with BUN at 29.2 mmol/L and creatinine at 122 μmol/L. The patient responded to active treatment, with sputum cultures returning negative on both July 1 and July 6. The procalcitonin (PCT) level decreased to 0.636 ng/mL on July 4. Polymyxin B was continued until July 12. Changes in renal function are shown in Figure 1.

Due to being medically unfit for surgical intervention, the patient was transferred to the Intensive Care Unit (ICU) on July 12 for life support and integrated treatment. The patient’s renal function was normal with the BUN 7.6 mmol/L and Cr 85 μmol/L on July 17. The ventilator was removed on July 28. The patient remained sedated and lethargic, with stable vital signs, but the overall condition was complex requiring systematic treatment. The family requested transfer to a local hospital for continued care.

## 3 Analysis and discussion

Sepsis is the leading cause of death in burn patients (9). With the progression of post-burn time, the incidence of Gram-negative bacterial infections increases, accompanied by a rising risk and prevalence of infections caused by multidrug-resistant organisms (10). This patient presented with multiple high-risk factors for drug-resistant Gram-negative infection, including extensive burn area, prolonged post-burn interval, mechanical ventilation, and a history of hospitalization at another institution. Considering this elevated risk, anti-infective therapy with meropenem and polymyxin B was initiated. Multiple cultures from secretions, sputum, and bronchoalveolar lavage fluid grew carbapenem-resistant *Acinetobacter baumannii* (CRAB), then the anti-infective regimen was adjusted to polymyxin B (100 mg IV q12h) and tigecycline (50 mg IV q12h), with the creatinine 59 μmol/L on June 25. Subsequently, the patient’s BUN and creatinine gradually increased. The serum creatinine rose to 149 μmol/L on June 27 and further increased on June 28. On June 29, the dosage of polymyxin B was adjusted to 50 mg every 12 h, and the patient’s blood urea nitrogen and creatinine levels then decreased. According to the Kidney Disease Improving Global Outcomes (KDIGO) diagnostic criteria for AKI (11), the patient’s creatinine increased by more than 26.5 μmol/L within 48 h, meeting the criteria for AKI. Late AKI in burn patients (≥48 h after burn injury) is associated with drug nephrotoxicity and sepsis (12). Using the Naranjo Adverse Drug Reaction Probability Scale, the score was 5 points, indicating a probable association with polymyxin B.

While polymyxin B-associated AKI has been reported, few studies have documented real-time dose-dependent renal function changes in severely burned patients with sepsis. In this case, the temporal relationship between dose escalation (from 50 to 100 mg q12h) and creatinine rise (from 59 to 163 μmol/L within 3 days), followed by renal recovery after dose reduction (to 50 mg q12h) without discontinuation, provides strong supportive evidence of causality. The patient was admitted due to burns and sepsis, but the serum creatinine level was normal. Acute kidney injury (AKI) occurred during the process of sepsis control (with a decrease in PCT). The patient had normal urine output (>4,000 mL/day) and no evidence of hypoperfusion. Furthermore, tigecycline rarely causes nephrotoxicity, and no aminoglycosides or vancomycin were used. Alternative causes of AKI, including burn-induced inflammation, sepsis, hypovolemia, and concomitant nephrotoxic agents, were systematically excluded.

Polymyxin B is commonly used clinically to treat infections caused by carbapenem-resistant Gram-negative bacteria (8, 13). The initial dose for this patient followed the conventional dosage recommended in the drug labeling. Due to repeated detection of CRAB and recurrent fever in the patient, and considering the patient’s body weight and pathophysiological condition, the dosage of polymyxin B was adjusted to 100 mg every 12 h on June 25. According to the international consensus guidelines and Chinese expert consensus on polymyxins, the recommended maintenance dose of polymyxin B for patients with normal renal function is 2.5–3 mg/(kg·d) (13, 14). After adjustment, this patient’s daily maintenance dose was 2.53 mg/kg, which aligns with these recommendations.

The primary adverse reaction of polymyxin B is kidney injury, which may manifest as elevated serum creatinine levels, casts in urine, proteinuria, or oliguria (15). Reported incidence rates vary widely across studies, ranging from 3–4% to as high as 60% (16–18), potentially due to differences in dosing regimens and patient baseline conditions. AKI can occur from 1 to 14 days after initiation, with the average time to onset typically being around the 4th day (18–20).

Regarding the risk factors for kidney injury induced by polymyxin B, findings vary across different studies. Potential risk factors include baseline serum creatinine levels, administration of a loading dose, trough concentration of polymyxin B, hypoalbuminemia, pre-existing chronic kidney disease, concurrent use of vasoactive agents, and concomitant use of other nephrotoxic drugs (18–22). This patient had no history of chronic kidney disease, with normal creatinine before dose adjustment, albumin levels above 30 g/L, no use of vasoactive agents or nephrotoxic drugs like aminoglycosides or vancomycin, but received a daily dose exceeding the labeled recommendation. Regarding the relationship between the daily dose of polymyxin B and AKI, research findings are inconsistent. However, most studies suggest that a higher daily dose is a risk factor for the development of kidney injury (20, 21, 23, 24). Nelson et al. conducted a study involving 151 patients treated with polymyxin B for bloodstream infections, which found that patients receiving doses <1.3 mg/(kg·d) had a significantly higher 30-day mortality rate, while a daily dose ≥250 mg was identified as an independent predictor for the development of AKI (OR = 4.32; 95% CI: 1.15–16.25) (23). A multicenter prospective cohort study by Rigatto et al. (24) showed that among patients treated with daily doses of polymyxin B < 150 mg, 150–199 mg, and ≥200 mg, the incidence rates of AKI were 32, 54, and 44%, respectively. Multivariate analysis indicated that a daily polymyxin B dose ≥150 mg was a risk factor for AKI (adjusted HR = 1.95, 95% CI: 1.31–2.89), while a daily dose ≥200 mg did not confer additional risk (24). A multicenter retrospective cohort study by Chang et al., which included 251 patients, found a higher proportion of patients exceeding the recommended daily dose in the AKI group compared to the non-AKI group (14.3% vs. 4.2%), however, further analysis did not find an independent correlation between the daily dose, cumulative dose, or duration of polymyxin B therapy and the occurrence of AKI (22). More research is needed to better assess and investigate the risk of kidney injury during clinical polymyxin B therapy and to optimize its clinical dosing strategies.

A limitation of this report is the absence of therapeutic drug monitoring (TDM) for polymyxin B, which would have allowed direct correlation between drug exposure and nephrotoxicity. TDM was not available at our institution during the patient’s hospitalization. Nevertheless, the temporal relationship between dose adjustment and renal function changes, together with the exclusion of other AKI causes, supports a probable causal association. In future practice, we recommend that burn patients receiving polymyxin B undergo TDM when feasible.

## 4 Conclusion

In summary, individualized treatment should be administered for patients receiving polymyxin B, based on factors such as the patient’s weight, pathophysiological condition, and infection status, aiming to optimize the regimen, avoid concurrent use of other nephrotoxic drugs whenever possible, analyze potential patient risk factors, enhance clinical monitoring, and conduct therapeutic drug monitoring when necessary. For patients at risk of kidney injury, such as burn patients in this case, urine output and urinalysis should be monitored during medication. Serum creatinine and BUN should be checked within 3 days after initiation to promptly identify and manage potential kidney injury, ensuring medication safety in clinical practice.

## Data Availability

The original contributions presented in the study are included in the article/supplementary material, further inquiries can be directed to the corresponding author.
